# Ecological analysis and environmental niche modelling of *Dactylorhiza hatagirea* (D. Don) Soo: A conservation approach for critically endangered medicinal orchid

**DOI:** 10.1016/j.sjbs.2021.01.054

**Published:** 2021-02-01

**Authors:** Ishfaq Ahmad Wani, Susheel Verma, Shazia Mushtaq, Abdulaziz Abdullah Alsahli, Mohammed Nasser Alyemeni, Mohd Tariq, Shreekar Pant

**Affiliations:** a Department of Botany, School of Biosciences and Biotechnology, Baba Ghulam Shah Badshah University, Rajouri 185234, J&K, India; b Department of Botany, S.P. College, Srinagar, Jammu and Kashmir, India; c Botany and Microbiology Department, College of Science, King Saud University, Riyadh, Saudi Arabia; d Meerut Institute of Engineering and Technology, Meerut, India

**Keywords:** *Dactylorhiza hatagirea*, Critically endangered, Conservation, Populations, Maximum entropy, Reintroduction, AUC, Area Under Curve, ROC, Receivers Operating Characteristic curve, ASCII, American Standard Code for Information Interchange, M. Gao, Masjid Gao, K.W.M, Kargil War Memorial, Asp, Aspect, Den, Density, Fre, Frequency, Ab, Abundance, D.P, Distribution pattern, Co, Contagious, Re, Regular, Ra, Random, SW, South-West, SE, South-East, NE, North-East, NW, North-West, E, East, m.a.s.l., meter above sea level

## Abstract

The natural populations of *Dactylorhiza hatagirea* have been greatly affected due to incessant exploitation. As such, studies on its population attributes together with habitat suitability and environmental factors affecting its distribution are needed to be undertaken for its conservation in nature. Present study aimed at accessing an impact of anthropogenic pressure on population structure and locate suitable habitats for the conservation of this critically endangered orchid. Considerable changes in the phytosociological attributes were observed on account of the changing magnitude and extent of anthropogenic threat in their natural abode. The distribution pattern of species indicated that more than 90% of the populations exhibit substantially aggregated spatial distribution. Maximum Entropy (MaxEnt) distribution modelling algorithm was used to predict suitable habitat and potential area for its cultivation and reintroduction. Twenty-seven occurrence records, nineteen bioclimatic variables, altitude, and slope were used. MaxEnt map output gave the habitat suitability for this species and predicted its distribution in the North-Western Himalayas of India for approximately 616 km^2^. Jackknifing indicated that maximum temperature of warmest month, annual mean temperature, mean temperature of the driest quarter, and mean temperature of the wettest quarter were the governing factors for its distribution and hence, presented a higher gain with respect to other variables. According to permutation importance, precipitation seasonality and mean temperature of wettest quarter shows the prominent impact on the habitat distribution. Results of AUC (area under curve) were statistically significant (0.940) and the line of predicted omission falls very close to an omission on training samples, validating a better run of the model. Response curves revealed a probable increase in the occurrence of *D. hatagirea* with an increase in mean temperature of the wettest quarter and maximum temperature of the warmest month contributed more than 50% to predicted habitat suitability. Direct field observations concurrent with predicted habitat suitability and google-earth images represent greater model thresholds for successful inception of the species. Together, the study proposes that the species can be conserved in or near its present-day natural habitats and is equally effective in determining the possible habitats for its cultivation and reintroduction.

## 1 Introduction

The driving factors that contribute to a decline in the distribution range and abundance of plant species in nature are over-exploitation, destruction of ecosystems, small and isolated populations, and unrestrained illegal activities (Rawat and Agarwal, 2015; Bachman et al., 2019, Lughadha et al., 2020). These interventions have caused almost an end to one-fifth of the plant species (Brummitt et al., 2010) and have dragged a greater portion of plants to different categories of threat (IUCN, 2016). Such alterations create serious obstacle for conservation biologists to develop effective strategies to address the conflicting demands between sustaining productivity and conserving biodiversity (Singh et al., 2017). Predicting and mapping the geographical locations and the boundaries of suitable ecological niches for the survival of a species forms a baseline in ecology and conservation as it helps to identify critical regions that may either need conservation action or protection (Warren and Seifert, 2011). These ecological parameters provide comprehensive details regarding dissemination of suitable ecosystems for the reintroduction of species and help to periodically track the growth parameters of plant species in their natural habitats for their effective restoration and protection (Gaston, 1996, Nazeri et al., 2010, Rodriguez-Salinas et al., 2010, Polak and Saltz, 2011).

The use of ecological niche modelling in predicting the corners of niche distribution for plant species recovery and reintroduction serves as a powerful approach and plays an important role in conservation (Ferrier, 2002; Kumar and Stohlgren, 2009, Adhikari et al., 2012, Sharma et al., 2018). It acts as the flourishing technique of ecological engineering to improve the populations of reduced organisms, their destroyed niches, and ecosystems (Ren et al., 2009, Zai et al., 2009). Recent advances in ecological niche modelling methods offer an unparalleled opportunity to forecast trends of species geographic distribution (Thullier et al., 2005, Peterson et al., 2007, Lopez-Darias et al., 2008, Peterson and Nakazawa, 2008). The ecological niche model-based predictions have been successfully used to combine species physiological threshold with remote sensing data and land cover to model and forecast sites that reflect the plant species' potential suitable habitats (Byers et al., 2002, Guisan and Thuiller, 2005, Aragón et al., 2010). The Maximum Entropy (MaxEnt) model is the most important statistical method for ecological modelling that helps to determine suitable habitat and future distribution area of plant species (Jaynes, 1957). It is quite a worthy database for precisely predicting species distribution as it utilizes entropy as a metric to extrapolate precise positions of the existence of the species. It does not require the inclusion of absence points based on a logical basis (Peterson and Soberon, 2012). MaxEnt modelling is favored over various statistical instruments because it only includes data on geographical coordinates and environmental variables (Elith et al., 2011, Phillips and Dudik, 2008) to assess the relationships between different variables by the use of categorical and continuous data (Phillips et al., 2006, Fuller et al., 2012). MaxEnt combines environmental variables with occurrence data, thereby generating a map showing the possible dispersion and distribution of species with different areas representing separate or similar suitability levels for each species (Chen and Peterson, 2000, Morrison and Hall, 2002).

*D. hatagirea* is a critically endangered medicinal orchid, inhabiting temperate to alpine regions at an elevation of 2500–5000 m.a.s.l (Wani et al., 2020). Owing to its medicinal value, it has got high importance in traditional (Ayurveda, Unani, and Siddha) as well as modern-day systems of medicine (Popli, 2017, Wani et al., 2020). The ever increasing demand for the species has resulted in its over-exploitation and unlawful trading, thus leading to population decline in nature (Uniyal et al., 2002). The species is listed under Appendix II of the Convention of International Trade in Endangered Species (Sharma et al., 2005). The study of its phytosociological characters and recognition of appropriate natural territory holds a rational stride for its conservation (cultivation and reintroduction). Taking into account the lack of this knowledge, current study is intended to identify potential habitat suitable areas in the North-western Himalayas of India and to improve understanding of the environmental factors that assess the suitability of their habitats, thus leading to better conservation efforts. The objectives of this research were to:

1. Study the distribution of *D. hatagirea* in natural habitats towards devising efficient management and conservation policies given the magnitude of threat to this high-value orchid;
2. Create an effective habitat model using Maximum Entropy (MaxEnt) species distribution tool and presence-only data;
3. Identify the role of different environmental variables in governing habitat suitability through ecological niche model-based analysis;
4. Undertake comprehensive field surveys to assess and associate population status with model thresholds in projected model niches;
5. Formulate conservation planning guidelines, identifying the role of aboriginal people in management activities, and directing areas for further study.

The present study provides a detailed evaluation of the population structure and habitat suitability of this globally endangered species. The use of publicly available data on bioclimatic variables and software in this study would make it possible for conservation biologists and national authorities to perform repeated in-country evaluations for effective management of this medicinal plant.

## 2 Materials and methods

### 2.1 Study area and ecological analysis

The study was undertaken in the erstwhile Jammu & Kashmir State and present Jammu and Kashmir and Ladakh Union Territories (UTs) from May 2015 to September 2019. Random field visits were performed and twenty-seven new populations were located from 13 different areas between the altitudinal range of 2231 and 3525 m.a.s.l. (avg. 3065.80 SD ± 216.12). Geographical coordinates, aspect, and altitude were recorded by Magellan Professional Mobile Mapper (990603-50). Occurrence points were therefore used as a habitat representative for *D. hatagirea* and subjected to further analysis. The quadrant approach was used to collect data on different population attributes, such as density (D), frequency (F), abundance (A), and species distribution patterns, following the outline of Kershaw (1973). Pearson’s correlation coefficient analysis was performed in order to study the effect of temperature and altitude on the distribution and numerical strength of *D. hatagirea.* The species distribution pattern was determined on the basis of abundance to frequency ratio. Value of A/F < 0.025 between 0.026–0.050 and >0.050 indicated regular, random and contiguous type of distribution respectively (Lomonilo, 2001).

### 2.2 Threat assessment

Information gathering from indigenous people by framing the questionnaire was undertaken to assess the threat to the *D. hatagirea* populations. Taking all possible stress (natural and anthropogenic) factors on board, a questionnaire was designed to gather information about the types of threats faced by *D. hatagirea*. Data was gathered from 250 respondents from different areas of the Ladakh region. Finally, the fully-filled questionnaires were analyzed and the feedback received was compiled to determine the different types and magnitude of the threat to the species.

### 2.3 Ecological niche modelling

The ecological niche modelling of *D. hatagirea* was performed using MaxEnt. Maximum entropy-based software, MaxEnt, estimates the likelihood of distribution of a species. MaxEnt requires the presence of environmental constraints (Phillips et al., 2006). Geographical coordinates of study sites were recorded and subjected to the modelling procedure. Nineteen environmental variables as potential predictors were considered for the ecological niche modelling of the species. These constraints were chosen on the basis of their biological significance for the disbursement of plant species and other environmental modelling studies.

### 2.4 Modelling procedure

Information of recorded geographical coordinates of 27 study sites was converted into degree decimal form (CSV format) for use as input to MaxEnt. In addition to species occurrence data, environmental data were also used as input for MaxEnt. Data of 19 bioclimatic variables that form environmental data were downloaded from the world climate data portal (http://www.worldclim.org). These bioclimatic variables represent limiting environmental factors, which include precipitation and temperature of the coldest or hottest month and annual trends and seasonality such as precipitation, annual temperature range, and mean etc. (Table 1).

**Table 1:** List of different bioclimatic variables used in ecological niche modelling.

Variable	Description	Temporal scale
Bio 1	Annual Mean Temperature	Annual
Bio 2	Mean Diurnal Range	Variation
Bio 3	Isothermality	Variation
Bio 4	Temperature Seasonality	Variation
Bio 5	Maximum Temperature of the Warmest Month	Month
Bio 6	Minimum Temperature of the Coldest Month	Month
Bio 7	Temperature Annual Range	Annual
Bio 8	Mean Temperature of Wettest Quarter	Quarter
Bio 9	Mean Temperature of Driest Quarter	Quarter
Bio 10	Mean Temperature of Warmest Quarter	Quarter
Bio 11	Mean Temperature of Coldest Quarter	Quarter
Bio 12	Annual Precipitation	Annual
Bio 13	Precipitation of Wettest Month	Month
Bio 14	Precipitation of Driest Month	Month
Bio 15	Precipitation Seasonality	Variation
Bio 16	Precipitation of Wettest Month	Quarter
Bio 17	Precipitation of Driest Month	Quarter
Bio 18	Precipitation of Warmest Quarter	Quarter
Bio 19	Precipitation of Coldest Quarter	Quarter

Source Hijmans et al. (2005).

Freely available 30 arc-seconds resolution data was downloaded and used (Scheldeman and Zonneveld, 2010). ‘GRID’ is the in-built format of these files and was converted to “ASCII” using Arc GIS 9.3 version so that data is made compatible with MaxEnt (Fielding and Bell, 1997). Shuttle Radar Topography Mission (SRTM) Digital Elevation Model (DEM) data obtained from (http://srtm.usgs.gov/index.php) was the source of elevation and slope.

### 2.5 Model calibration and validation

The model run performance was checked by perforating 25 model run replicates at the 10 percent training presence threshold rule. Model run replicates have been subjected to cross-validation for test results. For training and test results, MaxEnt generates Receiver Operating Curves (ROC). The area under curve (AUC) predicts the efficiency of a model (Phillips et al., 2006, Phillips and Dudik, 2008). Therefore, greater AUC values are believed to indicate good performance of the model run. AUC values above 0.75 are theoretically useful and are supposed as good predictors of habitat suitability while AUC values <0.7 as bad descriptors (Swets, 1988, Elith et al., 2011). Values ranging from 0.7 to 0.9 show fair predictive abilities and >0.9 act as strong descriptors. AUC values range between 0 and 1, with 0.5 as a random prediction.

### 2.6 Exploration of bioclimatic variables and their correlation

The variable contribution is measured at the training and testing phases of the model, with each variable omitted in turn or used separately giving an indicator of the 'value' or knowledge given to the model by each variable. In order to approximate variable value, jackknife simulations were performed. Contributions of different variables is rank-based in relation to the ordering of predictions; thus the procedure was replicated 25 times using bootstrap and the mean and range values were determined. In order to assess the possible overall relevance of different environmental variables for model forecasts, the average benefit from model repetitions was compared with 'with-only' AUC values to classify theoretically associated variables supplying the same model output information. Pearson correlation coefficients have been calculated between the variables. Variables displaying clustering and showing correlations >0.9 have been simplified to one variable by selecting the variable that gives the most importance to the model output.

### 2.7 Population status in relation to model thresholds

Large-scale field assessments and inventories were performed to transcend the reliability and significance of the model standards underlying the population status of the species in each occurrence area. Furthermore, the numerical intensity of the plant species (density) at various locations was then measured in the distribution models with the threshold values (very high, high, medium, and low). Populations bearing greater densities were superimposed with higher thresholds to approve habitat suitability for the reintroduction of species and vice versa (Adhikari et al., 2012).

### 2.8 Habitat status assessment for species reintroduction

**Fig. 1:**
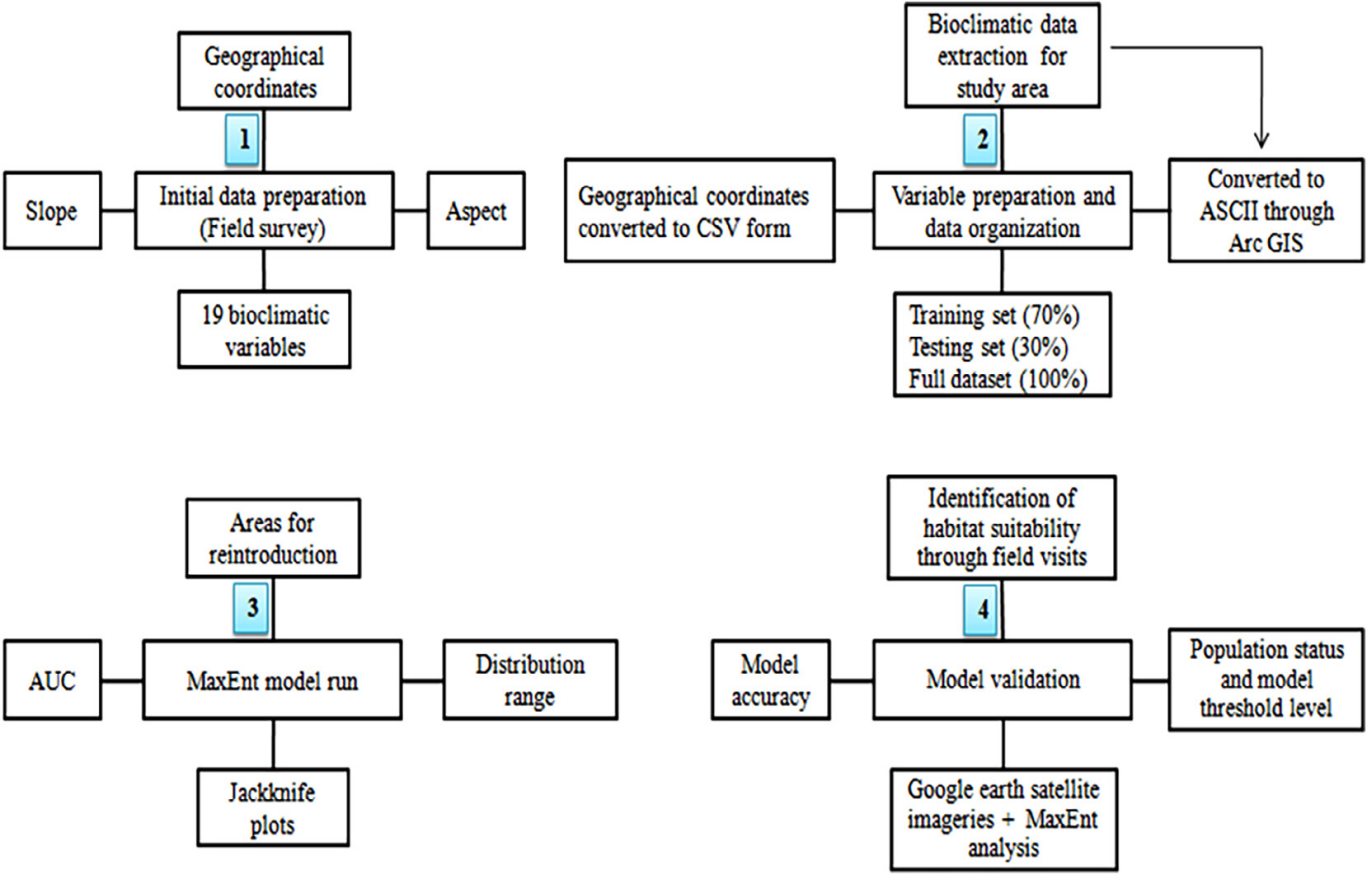
Flow chart of the methodology applied for determining the habitat suitability of *D. hatagirea*.

For determining the actual habitat conditions in the predicted suitable habitats, MaxEnt generated niche suitability map was changed to KMZ format using Diva GIS ver. 7.3 (www.diva-gis.org) and finally overlaid on Google Earth images. Repeated field surveys were performed on the basis of model production in the entire estimated potential region to determine the real habitat suitability. The approach used in assessing the suitability is shown in Fig. 1.

## 3 Results

### 3.1 Ecological analysis

A total of 110 populations (pre and post modelling) were inventoried from twenty-nine different locations from the entire Jammu and Kashmir and Ladakh UTs of India. Areas that were recognized as potentially suitable habitats were localized within an altitudinal gradient of 2231–3525 m.a.s.l. Sankoo has the highest number of populations (19) while Gundiyal, Dambur, Chutak, and Suktiyal have the lowest number of populations (1 each). More than 90% of the study areas reflect balanced population structure (better illustration of seedlings, saplings, young and adult individuals) and display a higher degree of regeneration. Due to grazing, trampling and other anthropogenic practices (overexploitation, vandalization, ecosystem fragmentation, and destruction), population characteristics such as density and abundance are dramatically impaired; while the frequency was least affected. Lowest density and abundance of *D. hatagirea* was reported from Sangrah 2 (0.15 and 3 ind/m^2^) and the highest was calculated for Purtikchey 1 (16.1 and 23 ind/m^2^). Frequency gives the distribution of *D. hatagirea* and ranged between 10% (Sangrah 1, Bihmbhat 1) and 90% (Masjid Gao 8, Masjid Gao 3, Sankoo 14). 89.09% of populations showed a contiguous type of dispersion which signifies the clumped or patchy species distribution pattern. Populations preferably show orientation in East, Southwest, and Southeast directions and combat Northeast and Northwest directions. Detailed results are provided in Table 2.

**Table 2:** Phytosociological attributes of *D. hatagirea* at North-Western Himalayas of India.

S.No.	Area	Altitude (m.a.s.l.)	Geographical Coordinates	Asp.	Den.	Freq.	Ab.	A/F	D.P
1	Bihmbhat1	3099	34°25′24.354°N 75°48′46.500° E	N.W	0.9	10	9	0.9	Co
2	Dras 1	3107.3	34°25′13.771° N 75°45′03.514° E	S.E	4.1	35	11.7	0.33	Co
3	Dras 2	3103.7	34°25′13.791° N 75°45′03.515° E	S.E	1.4	45	3.11	0.069	Co
4	Dras 3	3113.7	34°25′13.741° N 75°45′03.813° E	S.W	0.45	15	3	0.02	Re
5	K.W.M 1	3232	34°08′98.654° N 77°28′24.820° E	N.W	0.35	10	3.5	0.122	Co
6	K.W.M 2	3213.4	34°08′98.732° N 77°28′24.803° E	N.W	0.85	30	2.83	0.094	Co
7	K.W.M 3	3267.2	34°08′87.282° N 77°28′08.101° E	E	0.45	15	3	0.02	Re
8	K.W.M 4	3249.7	34°08′87.252° N 77°28′08.117° E	E	1.4	55	2.54	0.046	Ra
9	Kargee 1	3207.4	34°08′12.348° N 75°57′11.220° E	N.E	1.15	20	5.75	0.287	Co
10	Kargee 2	3234.5	34°06′19.818° N 75°57′07.944° E	S.W	2.45	30	8.16	0.272	Co
11	M. Gao 1	3084.7	34°25′30.750° N 75°45′42.144° E	S.W	15.25	85	17.94	0.211	Co
12	M. Gao 4	3083.5	34°25′30.749° N 75°45′42.143° E	E	0.6	15	4	0.240	Co
13	M. Gao 2	3079.9	34°25′30.757° N 75°45′42.132° E	E	11.75	90	13.05	0.145	Co
14	M. Gao 3	3073.3	34°25′30.768° N 75°45′42.121° E	S.W	13.20	90	14.66	0.162	Co
15	Panikhar 1	2367.8	34°06′44.040° N 75°57′05.346° E	S.E	5.35	25	21.4	0.856	Co
16	Purkichey 1	3228.4	34°08′56.574° N 75°57′02.474° E	E	14.05	80	17.56	0.219	Co
17	Purtikchey 1	3261	34°05′12.217° N 75°50′49.260° E	E	16.1	70	23	0.795	Co
18	Sangrah 1	2990	34° 15.991′ N 75°58.53′ E	N.W	0.25	10	2.5	0.25	Co
19	Sangrah 2	2982.3	34°17′12.732° N 75°15′11.142° E	N.E	0.15	15	3	0.2	Co
20	Sangrah 3	2977.2	34°17′12.742° N 75°15′11.132° E	N.E	0.45	25	1.8	0.072	Co
21	Sangrah 4	2979.8	34°17′12.740° N 75°15′11.135° E	S.E	0.65	35	1.85	0.053	Co
22	Sankoo 1	2979	34°17′21.359° N 75°57′51.887° E	S.W	2	30	10	0.33	Co
23	Sankoo 2	2981.7	34°17′23.347° N 75°57′50.292° E	E	5.75	45	12.77	0.28	Co
24	Sankoo3	3416	34° 16.467′ N 75°56.192′ E	E	0.45	20	2.25	0.11	Co
25	Thangbhoo	3202	34°12′09.960° N 75°55′56.568° E	E	9.4	80	11.75	0.146	Co
26	Nakpochu 1	3002	34°15′12.144° N 75°48′11.118° E	E	1.13	45	2.42	0.052	Co
27	Mulbekh 1	3021.4	34°55′39.071° N 76°13′49.101°E	S.W	6.43	70	9.19	0.13	Co

(*geographical coordinates from these sites were subjected to MaxEnt analysis).

Abbreviations: Asp. Aspect, Den. Density (ind/m^2^); Fr. Frequency (%); Ab. Abundance (ind/m^2^); D.P. Distribution Pattern; K.W.M. Kargil War Memorial; M.Gao. Majid Gao; Ra. Random; Re. Regular; Co. contiguous.

Altitude and temperature have a major influence on the ecological characters of *D. hatagirea*. While the rise in altitude has a negative influence, the increased temperatures have a positive effect on the population structure of *D. hatagirea*. Detailed findings as shown in Table 3.

**Table 3:** Effect of altitude and temperature on density and frequency of *D. hatagirea*.

Correlations		Altitude	Density	Abundance	Average temp (?C)
Altitude (m.a.s.l.)	Pearson Correlation	1	−0.351^**^	−0.316^**^	−0.526^**^
	Sig. (2-tailed)		0.002	0.005	0.000
	N		110	110	110

Density	Pearson Correlation		1	0.869^**^	0.228*
	Sig. (2-tailed)			0.000	0.046
	N			110	110

Abundance	Pearson Correlation			1	0.231*
	Sig. (2-tailed)				0.043
	N				110

**. Correlation is significant at the 0.01 level (2-tailed).

*. Correlation is significant at the 0.05 level (2-tailed).

N = Numer of sampling units.

### 3.2 Threat assessment

Regular field visits and a questionnaire designed for the indigenous people were chosen in assessing the threat to *D. hatagirea*. During the three-week-long evaluation process, 107 out of 250 respondents started to fill the questionnaires; however, only 73 of them were found completely filled and thus considered for analysis. Out of 73 respondents who filled the questionnaires, 35 were elderly male (>37 years), 21 were elderly female (>37 years) and 17 were teenagers. Analysis of the questionnaire revealed that 8% of respondents assume that the plants are being used to satisfy therapeutic requirements. They accepted that over the course of two decades, a significant number of populations that once displayed high abundance have decreased. Twenty-one percent of the respondents reported that grazers and tramplers pose a serious threat to the growth and population structure of the species as the above-ground parts are either trimmed or trampled and underground parts are either exposed or damaged. Nine percent of respondents highlighted habitat loss and deforestation due to unchecked anthropogenic activities (change in land-use practices and construction of infrastructure) as a reason for the reduction in *D. hatagirea* populations. Six percent of respondents proposed ornamental values of the species as a threat to the plant, while 27.8 percent and 3.3 percent of respondents reveal a lack of farming methods and inadequate irrigation systems as a possible source of threat to the plant. Overexploitation of the plant to feed livestock was a major concern among nineteen percent of the respondents and a fair number of respondents (5.4%) reported ignorance of the value of the species as a cause of threat too.

### 3.3 Model calibration

**Fig. 2:**
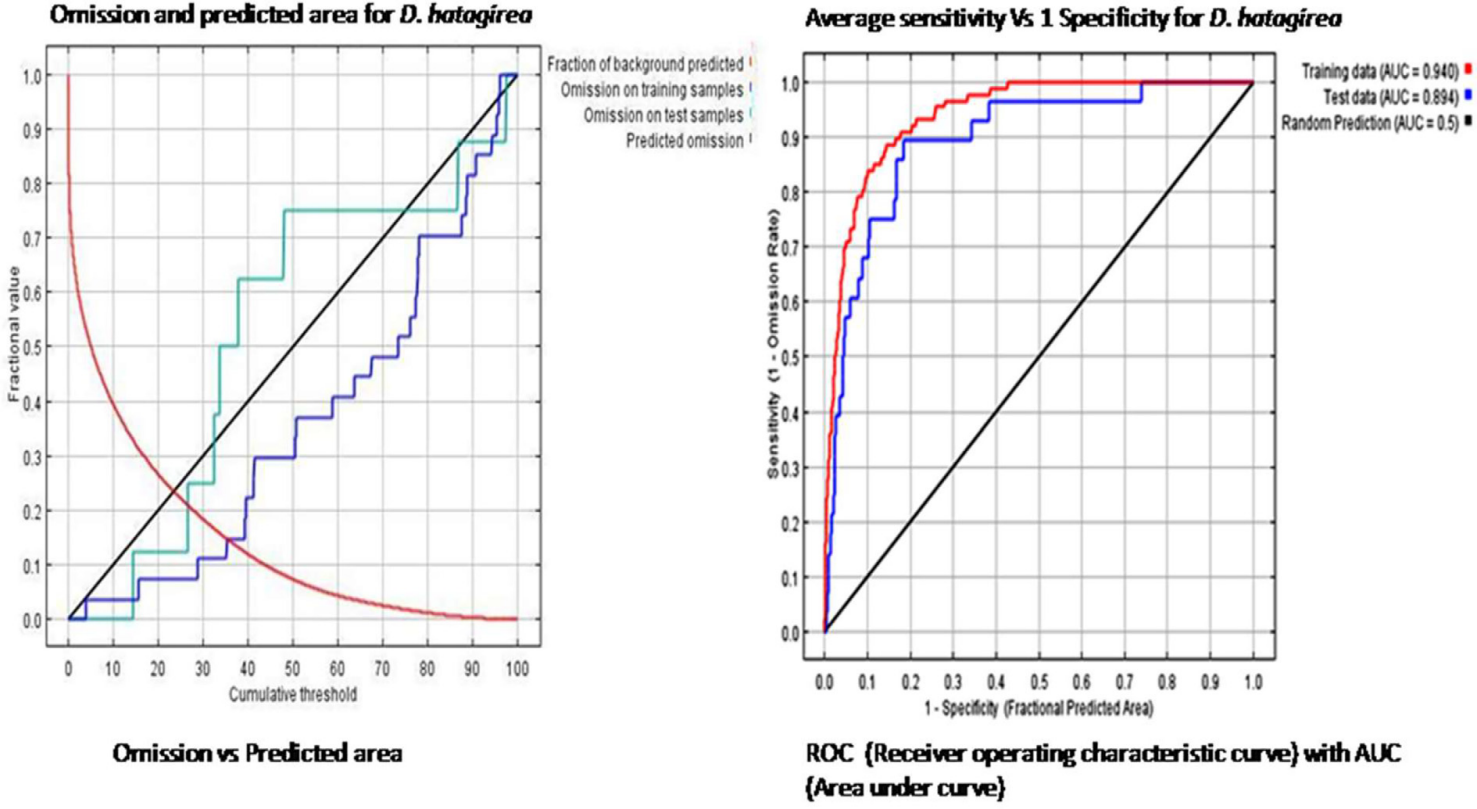
Representation of model calibration, **A**: Omission Vs predicted area **B**: ROC (Reciever Operating Characteristic curve) with AUC (Area Under Curve).

The omission rate and projected area is a function of the cumulative threshold. The rate of omission is calculated on training presence record and test record. The cumulative threshold means that the rate of omission should be close to the expected omission. In Fig. 2A black line indicates predicted emission, red line indicates the fraction of background predicted (mean area) and the blue line indicates omission on training samples. The line of predicted omission is very close to an omission on training samples (Fig. 2A). The mean value of AUC (area under ROC) curves obtained while developing a habitat suitability model of *D. hatagirea* was 0.940 i.e. close to 1 indicating that the model performed better representing the point of accuracy (Fig. 2B).

### 3.4 Potential habitat distribution map

**Fig. 3:**
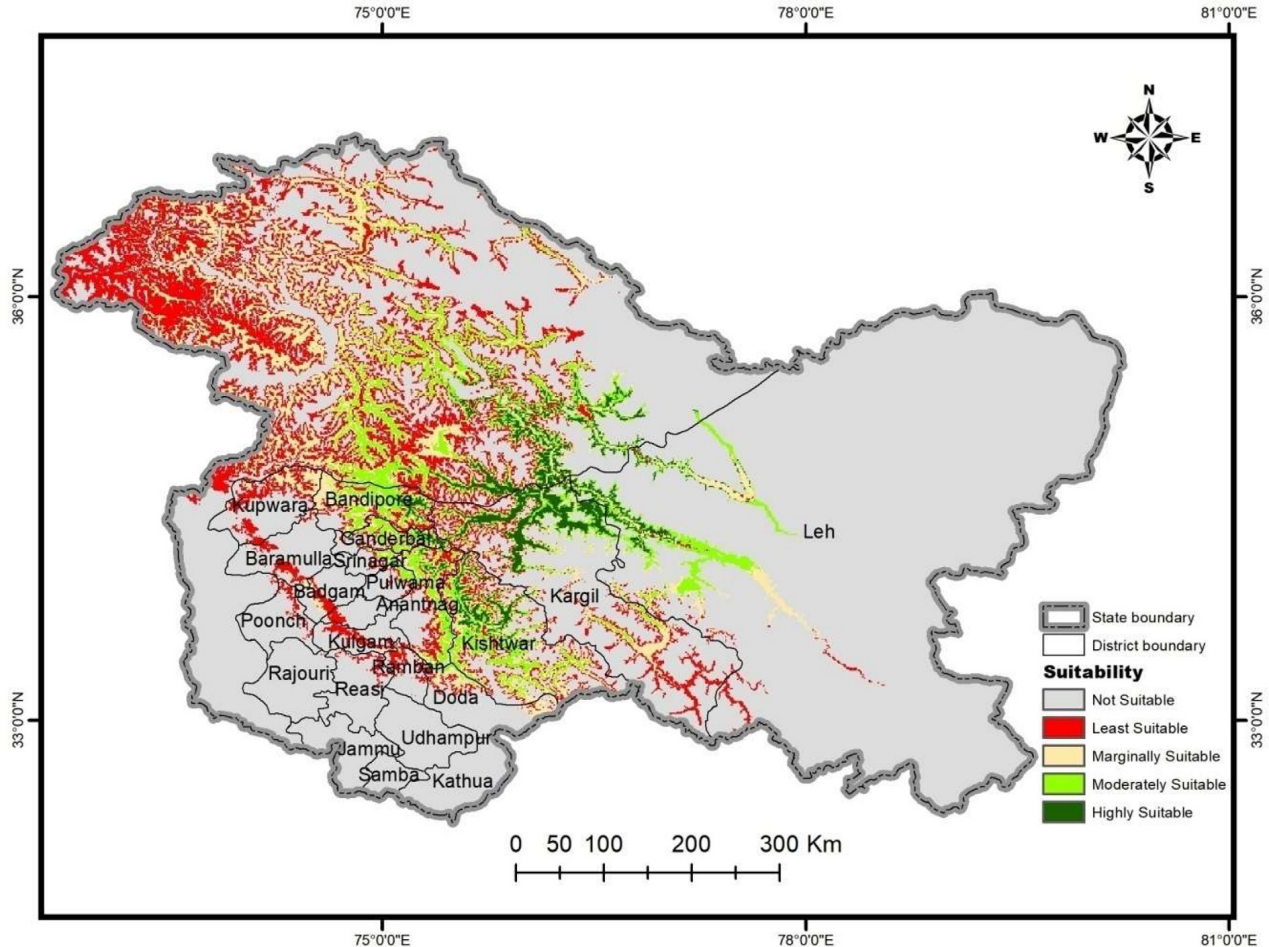
MaxEnt map for habitat suitability of *D. hatagirea* in the North-western Himalayas of India.

Habitat distribution modelling was performed for *D. hatagirea* in the North-Western Himalayas of Jammu and Kashmir and Ladakh UTs of India. Occurrence points for habitat distribution modelling were recorded between 2015 and 2019. MaxEnt model provided information about suitable areas with the best possible habitat for *D. hatagirea*. The most appropriate habitat and highest suitability thresholds for occurrence was predicted in the areas of Kargil, Dras, Bandipora, Sonmarg, Kishtwar and Doda districts. Some regions of District Banihal, Doda, Kishtwar, Pulwama, Anantnag, Ganderbal, Kargil, and Leh were also found to provide optimally suitable conditions for its distribution (Fig. 3).

**Fig. 4:**
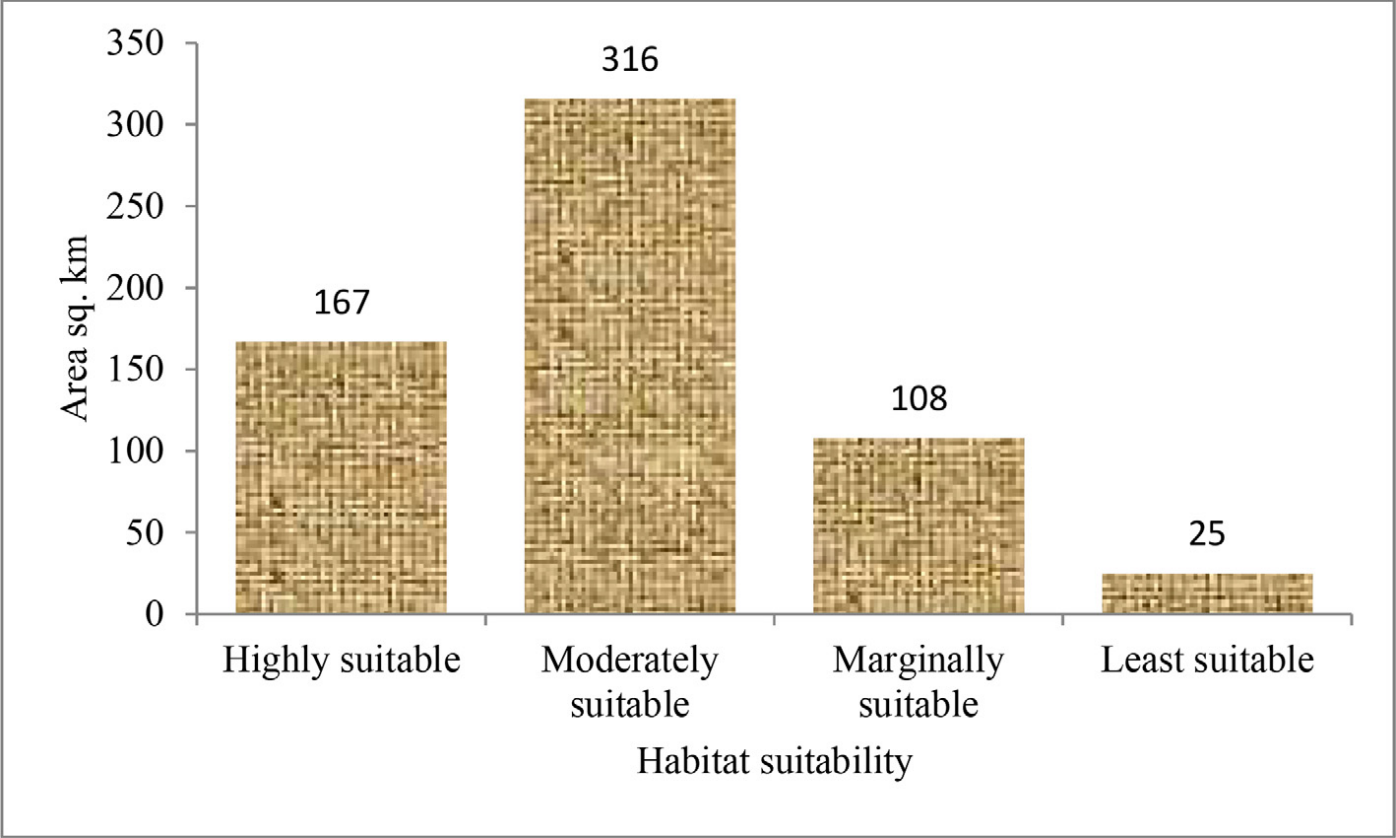
The area under different suitability grades for the optimal average model.

Based on the observations from primary field surveys, predicted suitable niches were mostly located in hillocks, sparse tree cover, protected grasslands, in and around human settlement areas, roadsides, and river banks. Degraded open forest lands, homestead gardens and human settlements were classified as areas of medium to low habitat suitability. Open grasslands, scrublands, and densely human settled areas were regions with very poor ecological suitability. A total potential area of ca. 300 km^2^ in the East and West of Northern Himalayas was predicted to be suitable for *D. hatagirea* reintroduction. Most of the areas fall under the medium suitability class and covers an area of 316 km^2^. Highly suitable areas were restricted to about 167 km^2^. 108 km^2^ was found to be marginally suitable while an area of low suitability was 25 km^2^ (Fig. 4).

### 3.5 Models internal jackknifing and response curves

**Fig. 5:**
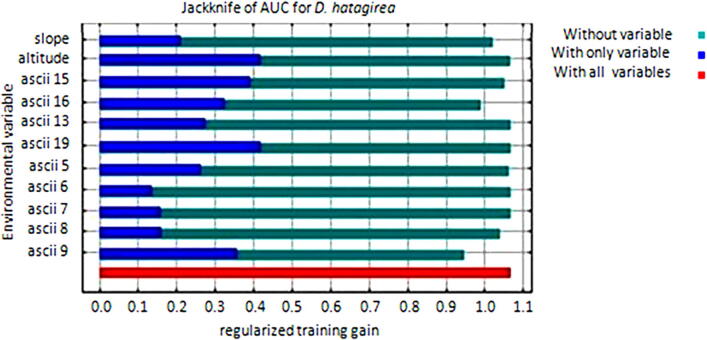
Jackknife evaluations result of the relative importance of predictive variables for MaxEnt model of *D. hatagirea*.

When a variable is omitted, a slight reduction in total benefit implies that other variables offer equivalent information, and removal of the variable does not result in a lack of predictive output. The model gain will determine the information each variable contributes to model output, variables that provide less information result in bad model gain and vice versa. Based on pearson's correlation, different bioclimatic variables that show clustering and correlations greater than 0.75 were reduced to one variable parameter by selecting the variable that provided maximum gain to model performance. Variable ascii 20 provides the most information or 'gain' as a single variable (with only), while ascii 15 decreases the gain when omitted (without), when compared to the model produced with all variables (Fig. 5).

**Fig. 6:**
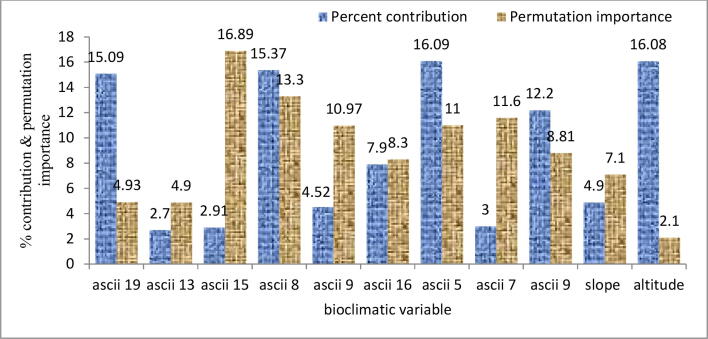
Graphical presentation of the overall mean percentage contribution and permutation importance of altitude, slope, and bioclimatic variables towards the development of the MaxEnt model through replicates of 25 model runs.

Models internal jackknifing reveals that elevation provides a significant role in governing the distribution of *D. hatagirea* and accounts for 16.08% of the total model run. Annual mean temperature (ascii 20), mean temperature of the wettest quarter (ascii 8), maximum temperature of the warmest month (ascii 5) and mean temperature of driest quarter (ascii 9) were the most influential and collectively contributed for 58.75% to the MaxEnt model. Rest of the six environmental variables account for 25% to the niche modelling of *D. hatagirea*. Considering the permutation importance, precipitation of driest quarter (ascii-18) presented greater influence on the habitat suitability and accounts for 46.5%, while rest of the environmental variables contributed to 53.5% of the total model run (Fig. 6).

**Fig. 7:**
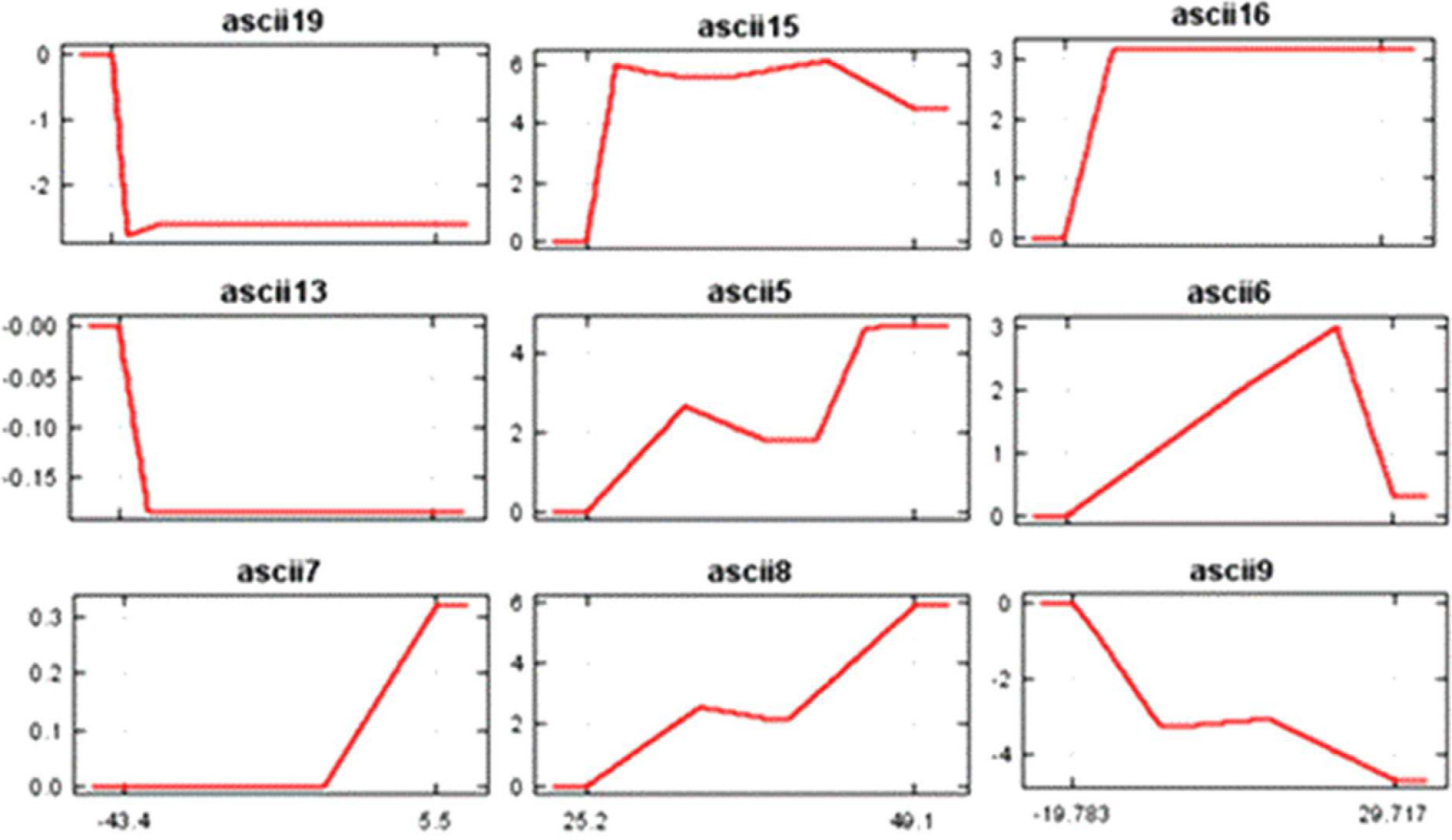
Response curves showing the effect of different bioclimatic variables on overall habitat suitability of D. *hatagirea.*

MaxEnt model curves generated give the dependence of habitat suitability on the selected variables and their correlations with different variables. Consequently the curves generated reveal that increase in mean temperature of the wettest quarter and maximum temperature of warmest month results in increased probability in an occurrence of *D. hatagirea* and decrease with increase in precipitation of driest month, precipitation of driest quarter (mm), precipitation of coldest quarter (mm) and precipitation of wettest quarter (Fig. 7).

### 3.6 Population status in relation to model thresholds and identification of areas for reintroduction

Direct field observations concurrent with model output reveal that a greater number of populations show better habitat suitability threshold level and fall under moderate to greater threshold categories. From the total suitable habitats located, 40.9% of populations show lower thresholds while the remaining 59.1% of populations were designated to be moderately or highly suitable. The major reason for lower thresholds being a serious anthropogenic threat received by the plant as recognized through questionnaire analysis. Google Earth satellite imageries equally benefits to the distribution pattern of *D. hatagirea* by providing similar results generated through MaxEnt analysis. Combined efforts from post modelling field surveys, Google Earth satellite imageries and the model output show that areas with high to very high habitat suitability for the species were disseminated to elevations ranging between 2800 and 3400 m.a.s.l. All these results were supported by the phytosociological attributes of the plant species as provided in Table 4.

**Table 4:** Phytosociological attributes of *D. hatagirea* in North-western Himalayas of India.

S.No	Area	Altitude (m.a.s.l.)	Geographical Coordinates	Asp.	Den.	Fre.	Ab.	A/F	D.P
1	Bihmbhat 2	3099	34°25′24.354° N 75°48′49.475° E	N.W	0.9	10	9	0.9	Co
2	Bihmbhat 3	2995	34°25′21.221° N 75°48′46.321° E	S.W	8.4	80	11.75	0.146	Co
3	Bihmbhat 4	3071	34°25′21.424° N 75°48′49.500° E	S.W	14.05	80	17.56	0.219	Co
4	Bihmbhat 5	2885	34°25′24.309° N 75°48′46.321° E	N.W	2.4	28	6.85	0.195	Co
5	Chiktan 1	3198	34°45′79.071°N 76°51′95.109°E	E	13.03	73	17.56	0.219	Co
6	Chiktan 2	3109.7	34°45′79.221°N 76°51′95.089°E	N.E	0.9	20	4.5	0.22	Co
7	Chiktan 3	3071.7	34°45′79.239°N 76°51′95.119°E	S.W	9.86	66	13.2	0.2	Co
8	Chuka 1	2985.9	34°17′23.724° N 75°57′50.796° E	S.W	14.06	75	19.18	0.261	Co
9	Chuka 2	3051	34°17′22.634° N 75°57′52.656° E	N.E	5.5	50	11	0.22	Co
10	Chukiyal 1	3090.6	34°25′26.703° N 75°50′20.376° E	N.E	1.3	30	4.33	0.144	Co
11	Chukiyal 2	2994	34°25′26.753° N 75°50′20.196° E	E	4.26	73.3	5.81	0.079	Co
12	Chukiyal 3	3106	34°25′25.474° N 75°50′20.866° E	S.W	0.76	56.6	1.35	0.023	Re
13	Chutak	2231	34°30′23.316° N 76°06′48.180° E	S.W	1.3	60	2.16	0.036	Ra
14	Dambur	2970	34°17′22.038° N 75°57′52.008° E	N.W	2.73	35	8.2	0.246	Co
15	Damsna 1	3196.9	34°10′19.458° N 75°54′55.818° E	N.W	2.4	35	6.85	0.195	Co
16	Damsana 2	3209	34°10′19.474° N 75°54′55.898° E	N.E	0.65	15	4.33	0.088	Co
17	Damsana 3	2967	34°10′20.221° N 75°54′56.286° E	S.E	8.1	80	10.12	0.126	Co
18	Dras 4	3087.5	34°25′13.823° N 75°45′03.397° E	E	1.55	40	3.87	0.096	Co
19	Dras 5	3121	34°25′04.157° N 75°45′03.215° E	N.W	0.8	30	2.66	0.08	Co
20	Dras 6	3127.7	34°25′02.573° N 75°45′93.197° E	N.W	2.35	50	4.7	0.1	Co
21	Garamthang1	2799.8	34°28′27.558° N 76°50′83.574° E	S.W	6.7	65	10.30	0.158	Co
22	Garamthang2	2865.7	34°28′27.578° N 76°50′83.539° E	S.W	8.46	70	12.09	0.172	Co
23	Gundiyal	2992	34°25′45.336° N 75°49′22.170° E	S.W	5.7	60	9.5	0.158	Co
24	K.W.M 5	3237.4	34°08′98.608° N 77°28′24.831° E	S.E	0.65	20	3.25	0.162	Co
25	K.W.M 6	3243.8	34°08′98.571° N 77°28′24.851° E	N.E	1.05	45	2.33	0.051	Co
26	K.W.M 7	3243.3	34°08′88.117° N 77°28′07.223° E	N.E	2.2	45	4.88	0.108	Co
27	K.W.M 8	3247.3	34°08′88.110° N 77°28′07.213° E	S.E	0.65	15	4.33	0.088	Co
28	Kargee 3	3183.7	34°08′12.388° N 75°57′11.272° E	S.W	1.15	20	5.75	0.287	Co
29	Kargee 4	3234.5	34°06′19.824° N 75°57′07.795° E	N.E	2.45	30	8.16	0.272	Co
30	Lamuchan 1	2809.1	34°25′43.938° N 75°47′34.788°E	N.W	3.9	70	5.57	0.079	Co
31	Lamuchan 2	3070	34°25′42.908° N 75°47′34.749° E	N.W	7.1	70	10.14	0.144	Co
32	Lamuchan 3	3051	34°25′42.944° N 75°47′34.818° E	N.W	0.9	20	4.5	0.22	Co
33	M. Gao 5	3086.4	34°25′30.747° N 75°45′42.147° E	S.W	8.1	80	10.12	0.126	Co
34	M. Gao 6	3085	34°25′30.748° N 75°45′42.145° E	N.E	1.35	55	2.45	0.044	Co
35	M. Gao 7	3097.4	34°25′30.617° N 75°45′42.235° E	N.E	0.95	15	6.33	0.422	Co
36	M. Gao 8	3079.9	34°25′30.617° N 75°45′42.132° E	N.E	1.65	90	1.83	0.020	Re
37	M. Gao 9	3061	34°25′30.775° N 75°45′42.108° E	N.W	0.45	15	3	0.2	Ra
38	M. Gao 10	3063.4	34°25′30.771° N 75°45′42.111° E	N.W	0.35	25	7	1.4	Co
39	Maita taisru	3253	34°07′30.366° N 75°55′53.670° E	E	6.35	55	11.45	0.209	Co
40	Manji 1	2755	34°29′04.118° N 76°06′12.918° E	N.E	1.3	50	2.6	0.052	Co
41	Manji 2	2766.9	34°29′06.204° N 76°06′14.514° E	N.W	1.5	40	3.75	0.093	Co
42	Mulbekh 2	2975	34°55′39.169° N 76°13′49.251°E	S.W	11.96	83.3	14.36	0.172	Co
43	Mulbekh 3	3005.1	34°55′39.178°N 76°13′49.237°E	S.W	1.15	20	5.75	0.287	Co
44	Nakpochu 2	2998	34°15′12.239° N 75°48′11.095° E	E	8.4	70	12.04	0.172	Co
45	Nakpochu 3	3091	34°15′13.479° N 75°48′11.981° E	N.E	0.93	56.6	1.55	0.027	Ra
46	Nakpochu 4	3107	34°15′13.321° N 75°48′11.671° E	N.E	0.45	20	2.25	0.11	Co
47	Panikhar 2	2769	34°07′08.442° N 75°57′06.254° E	S.W	15.1	83	18.12	0.218	Co
48	Panikhar 3	2909	34°07′08.318° N 75°57′06.025° E	S.W	4.1	45	9.11	0.202	Co
49	Panikhar 4	2837	34°07′06.829° N 75°57′07.294° E	N.W	5.06	50	10.13	0.020	Re
50	Panikhar 5	2739	34°07′08.312° N 75°57′06.626° E	N.W	0.43	20	2.16	0.108	Co
51	Purkichey 2	2954.5	34°08′59.189° N 75°57′04.719° E	S.W	5.45	70	7.76	0.11	Co
52	Purkichey 3	3127.9	34°08′56.973° N 75°57′02.254° E	S.W	14.05	80	17.56	0.219	Co
53	Purtikchey 2	3039	34°05′12.217° N 75°50′49.260° E	S.E	16.1	45	35.77	0.795	Co
54	Ragdum 1	2911	34° 17.239′ N 75°58.181′ E	N.E	1.9	30	6.33	0.211	Co
55	Ragdum 2	3951	33° 016.48′ N 75°57.927′ E	N.E	2.2	55	4	0.072	Co
56	Sangrah 5	2986.7	34°17′12.725° N 75°15′11.149° E	N.W	0.65	35	1.85	0.053	Co
57	Sangrah 6	2976.9	34°17′12.741° N 75°15′11.135° E	N.W	0.45	20	2.25	0.112	Co
58	Sangrah 7	2976	34°17′12.744° N 75°15′11.130° E	N.W	0.35	35	1	0.028	Ra
59	Sankoo 4	2973.3	34°17′23.364° N 75°57′50.274° E	S.E	5.35	75	7.13	0.095	Co
60	Sankoo 5	3443	34° 16.83′ N 75°56.504′ E	S.W	0.75	15	5	0.33	Co
61	Sankoo 6	3525	34° 16.633′ N 75°56.754′ E	S.W	4	75	11.42	0.326	Co
62	Sankoo 7	3034	34° 17.011′ N 75°57.227′ E	S.W	2.25	80	2.81	0.035	Ra
63	Sankoo 8	3034	34° 17.168′ N 75°57.227′ E	S.E	4.25	60	7.08	0.118	Co
64	Sankoo 9	2996	34° 17.168′ N 75°57.541′ E	N.E	3	60	5	0.083	Co
65	Sankoo 10	2974	34° 17.353′ N 75°57.729′ E	N.E	2.45	45	5.44	0.120	Co
66	Sankoo 11	2961	34° 17.381′ N 75°57.984′ E	N.E	1.75	15	11.66	0.77	Co
67	Sankoo 12	2948	34° 17.387′ N 75°57.998′ E	N.W	3	50	6	0.12	Co
68	Sankoo 13	3018	34° 16.958′ N 75°57.821′ E	N.W	5.5	85	6.47	0.076	Co
69	Sankoo 14	3044	34° 16.727′ N 75°57.818′ E	N.W	9.25	90	9.25	0.092	Co
70	Sankoo 15	2971.4	34°17′23.373° N 75°57′50.265° E	S.E	4.1	45	9.11	0.202	Co
71	Sankoo 16	2979.4	34°17′23.351° N 75°57′50.287° E	S.E	10.65	75	14.2	0.189	Co
72	Sankoo 17	2977	34°17′23.354° N 75°57′50.281° E	S.W	1.35	55	2.45	0.044	Ra
73	Sankoo18	2980	34°17′21.354° N 75°57′51.900° E	S.W	1	20	5	0.25	Co
74	Sankoo 19	2982	34°17′21.344° N 75°57′51.345° E	S.W	1.75	50	7.5	0.15	Co
75	Suktiyal	3124	34°25′46.680° N 75°45′36.006° E	E	1.3	50	2.6	0.052	Co
76	Thangbhoo 2	3107	34°12′09.960° N 75°55′56.568° E	E	9.4	80	11.75	0.146	Co
77	Thangbhoo 3	3252	34°07′06.716° N 75°52′51.168° E	N.E	5.2	55	9.45	0.171	Co
78	Thasgam	3011.8	34°17′19.974° N 75°58′05.580° E	N.W	5.93	65	8.9	0.133	Co
79	Wakha 1	2754.4	34°22′24.265° N 76°23′13.474° E	S.E	1.3	60	2.16	0.036	Ra
80	Wakha 2	2698	34°22′24.321° N 76°23′13.531° E	S.E	2	30	10	0.33	Co
81	Wakha 3	2781.8	34°22′24.298° N 76°23′13.289° E	S.W	5.75	45	12.77	0.28	Co
82	Maita Taisru1	3109.3	34**°** 07′30.366′N 75**°** 55′53.670′E	E	2.45	40	5.5	0.11	Co
83	Maita Taisru 2	3209	34**°** 07′30.476′N 75**°** 55′53.783′E	E	9.4	80	11.75	0.146	Co

(* sites explored after MaxEnt and google earth superimposing).

Abbreviations: Asp: Aspect, Den: Density (ind/m^2^), Fre: Frequency (%), Ab: Abundance (ind/m^2^), DP: Distribution pattern.

## 4 Discussion

The ever-increasing demand for medicinal plants has led to over-exploitation and degradation of their natural habitats. It has resulted in reduced plant populations in native habitats that progressed to the extinction of several important plant species (Brummitt et al., 2010, Barnosky et al., 2011). As habitat protection and its enrichment became important towards conservation and rehabilitation of plants, inputs of ecological sciences and biogeography harnessed an understanding of the relationship between a particular plant species with respect to their surrounding environment (Guisan and Zimmerman, 2000, Franklin, 2009, Barik and Adhikari, 2011, Polak and Saltz, 2011). Remote sensing attributes of sensing input variables such as biome, landscape or eco-region maps, vegetation type, and the density (Turner et al., 2003, Kushwaha, 2011) with data integration, modelling, and creation of geospatial database using Geographic Information System (GIS), have helped in designing strategic management policies for their conservation (Irfan-Ullah et al., 2006).

As plants growing in diverse habitats have shown superiority over species with narrow habitat ranges, they exhibit wider distribution and experience a lesser risk of extinction (Shrestha and Bawa, 2014). From a conservation point of view, orchids face serious threat due to high medicinal values, human intrusion, habitat loss, deforestation, degradation, and overgrazing (Pant and Raskoti, 2013, Warghat et al., 2016). Although attempts towards conservation of *D. hatagirea* through ecological niche modelling has been initiated in the Himalayas of Nepal (Kunwar et al., 2020), however prior this study such studies were lacking in the North Western Himalayas of India. In two Union Territories (UTs; Jammu & Kashmir and Ladakh), *D. hatagirea* recorded serious threats in their natural habitats as revealed from the lower phytosociological attributes at various populations. Direct field observations and questionnaire analysis revealed that indiscriminate exploitation of the species to meet medicinal needs and uncontrolled grazing were the main reasons behind the decrease in their populations. Though the plant bears some degree of anthropogenic disturbance, however, the effect becomes prominent when the intensity of the alteration increases (Schmitz and Isselstein, 2020). A drastic decrease in the density and abundance was reported among severely affected populations compared to their counterparts. Such results are in accordance with the findings of Nautiyal et al., 2004, Bhatt et al., 2005, Uniyal et al., 2002, Sharma et al., 2005, Hijmans et al., 2005, Kala, 2005, Jalal and Rawat, 2009 who also reported a significant decrease in the phytosociological attributes of *D. hatagirea* on account of severe anthropogenic pressure at different locations. A significant proportion of populations (~95%) were found exhibiting aggregated distribution patterns. In accordance with the previously published records regarding the clumped distribution of *D. hatagirea,* this characteristic distribution pose a major threat by allowing an easy collection of the plant material by exploiters (Pillon et al., 2006).

For precisely predicting species distribution, maximum entropy (MaxEnt) represents a standard model (Phillips et al., 2006). Analysis and confirmation are the two ways through which the accomplishment of an ecological model can be achieved. Whether or not the given results adept through the model are accurate requires to be put to a test in the course of time. Therefore, an analysis of the threshold binomial test (omission/commission rate) based on omission and predicted area was carried out (Phillips and Dudik, 2008). A poor model has AUC near to 0; an area of 1 represents a perfect test; an area of 0.5 represents that the model has no class separation capacity whatsoever or the model is close to random and is a poor indicator (Swets, 1988). From the given model it is revealed that lines of omission from the training data are almost near to predicted omission and AUC value in case of training is 0.94 which is close to 1 and this points towards our model accuracy.

Direct field studies concurrent with the models internal jackknifing reveal constrained allocation of *D. hatagirea* to specific elevations. Such findings indicate near endemism of *D. hatagirea* to the Himalayan region. The use of bioclimatic variables through MaxEnt describes the role of environmental factors in determining habitat appropriateness of the species (Warren and Seifert, 2011, Ma and Sun, 2018). The greater contribution of ascii 20, ascii 5, ascii 8 and, ascii 9 among different bioclimatic variables show a vital role played by these factors in defining habitat suitability for *D. hatagirea*. Most importantly ascii 9 which contributed more to the habitat suitability is mainly associated with peak flowering and fruiting of *D. hatagirea*. This indicates that modelling through MaxEnt can act as a powerful and informative tool that can be used to determine the borders of the potential habitat of the species (Cao and Tang, 2014, Yi et al., 2016). Despite the fact that certain areas were predicted to be highly suitable for the habitat of this plant, timely field observations revealed different anthropogenic disturbances (trampling and grazing) as a driving force for the population degradation. Based on these interpretations, we can undertake that population structure of a species in unruffled niches within their local range could be affirmed via model output, i.e. locales accommodating larger population size be estimated as exemplars with a raised threshold level and vice versa (Adhikari et al., 2012, Adhikari et al., 2019).

ENM results strongly suggest it as a valuable tool for efficient *in situ* conservation by identifying the areas that contain patches of *D. hatagirea* populations. MaxEnt predicted that certain regions such as Kishtwar, Bandipora, Ganderbal, and Anantnag of Jammu and Kashmir can provide possible suitable habitat for the conservation of this species provided that adequate measures are taken to protect the habitat. The strong relationship between model thresholds and population size depicts the importance of ecological niche modelling in population studies. For the conservation of *D. hatagirea,* those areas that are depicted by MaxEnt output will not only help in the reintroduction of *Dactylorhiza* in areas where the species had existed before but also in enriching the species populations and improving its conservation status. Such results enable natural resource managers and conversation biologists in the efficient conservation and management of threatened taxa, including *D. hatagirea*, and conserving its overall genetic diversity in these regions.

## 5 Implications for conservation, community engagement, and future research

Phytosociological analysis based on field inventories facilitates improved characterization and efficient interpretation of the suitability of the environment while forecasting and plotting possible suitable habitats for this highly valuable and critically endangered species. This study offers valuable guidance in determining areas for undertaking future field studies, choosing reserves, and directing decisions on its environmental management. Based on the ecological analysis, the major factors that threaten the persistence of *D. hatagirea* are an uncontrolled anthropogenic disturbance. Additionally status of other unreported populations thriving in inaccessible areas as well as expansion of land conversion to agriculture there, a periodical revisit to population viability analysis needs to be a priority area in the future. Minimizing the magnitude and extent of all possible threats in natural habitat might be one of the basic strategies for preserving species that have got a high risk of extinction. The focus needs to be upon reduction of biotic pressure rehabilitation, an establishment of protected area network, with appropriate management practices, corridors to link fragments to restore degraded habitats. Promotion of *ex-situ* propagation in controlled environments such as natural habitats, botanic gardens and other conservation facilities could greatly aid in increasing the recovery rate of this important medicinal herb. Protecting populations in their natural habitat and restoring ecosystems require the participation of community, non-government organizations (NGO’s), educational, research institutions, and different government agencies. The establishment of bio-banks and cryo-conservation plants for the collection and preservation of specimens and genetic material can also be considered as potential *ex-situ* strategy for long term survival of the species. To reintroduce *ex-situ* raised plant material in natural habitats ecological niche modelling using occurrence records and multiple climatic variables is going to be extremely helpful. Moreover, to predict habitat loss due to climate change or land transfer, future use of this modelling approach should consider extrapolating habitat suitability under varying climatic conditions and integrating fine-scale mapping. Use of advanced biotechnological applications, such as high throughput genotyping and gene sequencing, metabolomics, Metagenomics, and transcriptomics should be preferred so that it allows genetic characterization of the plant which may lead to the taxonomic and evolutive characterization of this plant. Bioinformatics coupled with the above mentioned biotechnological tools allows to interpret and practice genotyping information and to accomplish bio-bank archives.

## 6 Conclusions

Current study defines the application of ecological niche modelling and population attributes in pointing out the areas that support *D. hatagirea* using sophisticated spatial resolution data, occurrence points, and environmental variables. This study provides the first predicted potential habitat distribution map for this species in North-west Himalaya of India which can assist in exploring new populations and developing better land-use regulation near their natural territories and for developing suitable conservation strategies for this species.

## Author contribution

SV conceived the idea; IAW performed the field work and contributed to the production of figures and tables; SV IAW & AAA contributed towards the analysis and interpretation of data and writing of the manuscript. IAW, MT, SP, SM & MNA contributed in MaxEnt analysis.

## Declaration of Competing Interest

The authors declare that they have no known competing financial interests or personal relationships that could have appeared to influence the work reported in this paper.

## References

[b0005] Adhikari D., Singh P.P., Tiwari R., Baril S.K. (2019). Modelling the environmental niche and potential distribution of *Magnolia campbelli* Hook.f. and Thomson for its conservation in eastern Himalaya. Pl. Comer. Val..

[b0010] Adhikari D., Barik S.K., Upadhaya K. (2012). Habitat distribution modelling for reintroduction of Ilex khasianaPurk., a critically endangered tree species of north eastern India. Ecol. Eng..

[b0015] Aragón P., Baselga A., Lobo J.M. (2010). Global estimation of invasion risk zones for the western corn rootworm: Diabrotica virgifera virgifera: integrating distribution models and physiological thresholds to assess climatic favourability. J. Appl. Ecol..

[b0020] Bachman S.P., Field R., Reader T., Raimondo D., Donaldson J., Schatz G.E., Lughadha E.N. (2019). Progress, challenges and opportunities for Red Listing. Biol. Conserv..

[b0025] Barik S.K., Adhikari D., Bhatt J.R., Singh J.S., Tripathi R.S., Singh S.P., Kohli R.K. (2011). Predicting geographic distribution of an invasive species *Chromolaena odorata* L. (King) & H.E. Robins. Invasive Alien Plants – An Ecological Appraisal for the Indian Sub-continent.

[b0030] Barnosky A.D., Matzke N., Tomiya S., Wogan G.O.U., Swartz B., Quental T.B., Marshall C. (2011). Has the earth’s sixth mass extinction already arrived?. Nature.

[b0035] Bhatt A., Joishi S.K., Garola S. (2005). *Dactylorhiza hatagirea* (D. Don) Soo- A west himalayan orchid in Peril. Curr. Sci..

[bib318] Brummitt N., Bachman S., Lughadha E.M.S. (2010). Plants under pressure a global assessment.. The first report of the IUCN Sampled Red List Index for Plants. Royal Botanic Gardens, Kew, UK..

[b0050] Byers J., Reichard S., Randall J.M., Hayes D. (2002). Directing Research to Reduce the Impacts of Nonindigenous Species. Conserv. Biol..

[b0055] Cao D.M., Tang W.C. (2014). Satellite-derived vegetation indices contribute significantly to the prediction of epiphyllous liverworts. Ecol. Ind..

[b0060] Chen G.J., Peterson A.T. (2000). A new technique for predicting distribution of terrestrial vertebrates using inferential modelling. Zool. Res*.*.

[b0065] Elith J., Phillips S.J., Hastie T., Dudik M., Chee Y.E., Yates C.J. (2011). A statistical explanation of Maxent for ecologists. Divers. Distrib..

[b0070] Ferrier S. (2002). Mapping spatial pattern in biodiversity for regional conservation planning: where to from here?. Syst. Biol..

[b0075] Fielding A.H., Bell J.F. (1997). A review of methods for the assessment of prediction errors in conservation models. Environ. Conserv..

[b0080] Franklin J. (2009). Mapping Species Distributions: Spatial Inference and Prediction.

[b0085] Fuller D.O., Ahumada M.L., Quinones M.L. (2012). Nearpresent and future distribution of Anopheles albimanus in Mesoamerica and the Caribbean Basin modeled with climate and topographic data. Int. J. Health Geogr..

[b0090] Gaston K.J. (1996). Species richness: measure and measurement. Biodiversity: a biology of numbers and difference.

[b0095] Guisan A., Thuiller W. (2005). Predicting species distribution: offering more than simple habitat models. Ecol. Lett.*.*.

[b0100] Guisan A., Zimmerman N.E. (2000). Predicting habitat distribution models in ecology. Ecol. Model..

[b0105] Hijmans R.J., Cameron S.E., Parra J.L., Jones P.G., Jarvis A. (2005). Very high resolution interpolated climate surfaces for global land areas. Int. J. Climatol..

[b0110] Irfan-Ullah M., Amarnath G., Murthy M.S.R., Peterson A.T. (2006). Mapping the geographic distribution of *Aglaiabour dillonii* Gamble (Meliaceae), an endemic and threatened plant using ecological niche modelling. Biodivers. Conserv..

[b0115] Jalal J.S., Rawat G.S. (2009). Habitat Studies for Conservation of Medicinal Orchids of Uttarakhand. Western Himalaya. Afr. J. Plant Sci..

[b0120] Jaynes E.T. (1957). Information theory and statistical mechanics. Phys. Rev..

[b0125] Kala C.P. (2005). Indigenous uses, population density, and conservation of threatened medicinal plants in protected areas of the Indian Himalaya. Conserv. Biol..

[b0130] Kershaw K.A. (1973). Quantitaive and Dynamic Ecology.

[b0135] Kumar S., Stohlgren T.J. (2009). Maxent modelling for predicting suitable habitat for threatened and endangered tree *Canacomyrica monticola* in New Caledonia. J. Ecol. Nat. Environ..

[b0140] Kunwar R.M., Rimal B., Sharma H.P., Poudel R.C., Pyakurel D. (2020). Distribution and habitat modelling of *Dactylorhiza hatagirea* (D.Don) Soo, *Paris polyphylla* sm. and *Taxus* species in Nepal Himalayas. J. App. Res. Med. Arom..

[b0145] Kushwaha S.P.S., Bhatt J.R., Singh J.S., Tripathi R.S., Singh S.P., Kohli R.K. (2011). Remote sensing of invasive alien plant species. Invasive Alien Plants – An Ecological Appraisal for the Indian Sub-continent.

[b0150] Lomonilo M.V. (2001). Elevation gradients of species density; historical and prospective views. Glob. Ecol. Biogeogr..

[b0155] Lopez-Darias M., Lobo J.M., Gouat P. (2008). Predicting potential distributions of invasive species: the exotic Barbary ground squirrel in the Canarian archipelago and the west Mediterranean region. Biol. Invasions..

[b0160] Lughadha E.N., Bachman S.P., Leao T.C.C., Forest F., Halley J.M. (2020). Extinction risk and threats to plants and fungi. Plants People Planet.

[b0165] Ma B., Sun J. (2018). Prediciting the distribution of *Stipa purpurea* across the Tibetean Plateau via the Maxent model. BMC Ecol..

[b0170] Morrison M.L., Hall L.S., Scott J.M., Heglund P.J., Morrison M.L., Haufler J.B., Raphael M.G., Wall W.A., Samson F.B. (2002). Standard terminology: toward a common language to advance ecological understanding and application. Predicting Species Occurrences: Issues of Accuracy and Scale.

[b0175] Nautiyal M.C., Nautiyal B.P., Prakash V. (2004). Effect of Grazing and Climatic Changes on Alpine Vegetation of Tungnath, Garhwal Himalaya, India. Environmentalist..

[b0180] Nazeri M., Jusoff K., Bahaman A.R., Madani N. (2010). Modelling the potential distribution of wildlife species in the tropics. World J. Zool..

[b0185] Pant B., Raskoti B.B. (2013). Medicinal orchid of Nepal. Himalyan Map House Kathmandu.

[b0190] Peterson T., Soberon S. (2012). Species Distribution Modelling and Ecological Niche Modelling: Getting the Concepts Right A. Brazilian J. Nat. Conserv*.*.

[b0195] Peterson A.T., Nakazawa Y. (2008). Environmental data sets matter in ecological niche modelling: an example with Solenopsis invicta and Solenopsis richteri. Glob. Ecol. Biogeogr..

[b0200] Peterson A.T., Papes M., Eaton M. (2007). Transferability and model evaluation in ecological niche modelling: a comparison of GARP and Maxent. Ecography.

[b0205] Phillips S.J., Anderson R.P., Schapire R.E. (2006). Maximum entropy modelling of species geographic distributions. Ecol. Model..

[b0210] Phillips S.J., Dudik M. (2008). Modelling of species distributions with Maxent: new extensions and a comprehensive evaluation. Ecography.

[b0215] Pillon Y., Ray M.F., Shipunov A.B., Chase M.W. (2006). Species diversity versus Phylogenetic Diversity: A Practical Study in the Taxonomically Difficult Genus *Dactylorhiza* (Orchidaceae). Biol. Conserv..

[b0220] Polak T., Saltz D. (2011). Reintroduction as an ecosystem restoration technique. Conserv. Biol.*.*.

[b0225] Popli D. (2017). Elicitation of Dactylorhin-E and Studying Anti-Cancerous Potential of *Dactylorhiza Hatagirea* D. Don; Dissertation submitted to Jay Pee.

[b0230] Ren H., Lu H., Shen W., Huang C., Guo Q., Li Z., Jian S. (2009). Sonneratia apetala Buch. Ham in the mangrove ecosystems of China: an invasive species or restora-tion species?. Ecol. Eng..

[b0235] Rodriguez-Salinas P., Riosmena-Rodriguez R., Hinojosa-Arango G., Muniz-Salazar R. (2010). Restoration experiment of Zostera marina L in a subtropical coastal lagoon. Ecol. Eng..

[b0240] Scheldeman X., Zonneveld M. (2010). Training manual on spatial analysis of plant diversity and distribution. Biodivers. Int. Rome..

[b0245] Schmitz A., Isselstein J. (2020). Effect of Grazing System on Grassland Plant Species Richness and Vegetation Characteristics: Comparing Horse and Cattle Grazing. Sustainability..

[b0250] Sharma P.K., Sharita S., Prell J. (2005). *Dactylorhiza hatagirea* (D. Don) Soo—A West Himalayan Orchid in Peril. Curr. Sci..

[b0255] Sharma S., Arunachalam K., Bhavsar D., Kala R. (2018). Modelling habitat suitability of *Perilla frutescens* with MaxEnt in Uttarakhand—A conservation approach. J. Appl. Res. Med. Arom. Plants..

[b0260] Shrestha U.B., Bawa K.S. (2014). Harvesters perceptions of population status and conservation of Chinese caterpillar fungus in the Dolpa region of Nepal. Reg. Environ. Change.

[b0265] Singh K.S., Sharma M., Pandey A. (2017). Biodiversity-Threats and Conservation..

[b0270] Swets J.A. (1988). Measuring the accuracy of diagnostic systems. Science.

[b0275] The IUCN Red List of Threatened Species. 2016. International Union for Conservation of Nature and Natural Resources (IUCN).

[b0280] Thullier W., Richardson D.M., Pysek P., Midgley G.F., Huges G.O., Rouget M. (2005). Niche-based modelling as a tool for predicting the risk of alien plant invasions at a global scale. Glob. Change Biol..

[b0285] Turner W., Spector S., Gardiner N., Fladeland M., Sterling E., Steininger M. (2003). Remote sensing for biodiversity science and conservation. Trends Ecol. and Evol..

[b0290] Uniyal S.K., Awasthi A., Rawat G.S. (2002). Current Status and Distribution of Commercially Exploited Medicinal and Aromatic Plants in Upper Gori Valley, Kumaon Himalaya, Uttaranchal. Curr. Sci..

[b0295] Wani I.A., Kumar V., Verma S., Jan A.T., Rather I.A. (2020). Dactylorhiza Hatagirea (D. Don) Soo: A critically endangered Perennial orchid from the North-West Himalayas. Plants.

[b0300] Warghat A.R., Bajpai P.K., Srivastava R.B., Chaurasia O.P., Sood H. (2016). Population genetic structure and conservation of small fragmented location of *Dactylorhiza hatagirea* in Ladakh region of India. Sci. Hortic..

[b0305] Warren D.L., Seifert S.N. (2011). Ecological niche modelling in Maxent: the importance of model complexity and the performance of model selection criteria. Ecol. Appl..

[b0310] Yi Y.J., Chenga X., Yanga Z.F., Shang-Hong Zhang S.H. (2016). Maxent modelling for predicting the potential distribution of endangered medicinal plant (H. riparia Lour) in Yunnan, China. Ecol. Eng..

[b0315] Zai X., Quin P., Wan S., Zhao F., Wang G., Yan D. (2009). The application of beach plum (*Prunus maritima*) to wasteland vegetation recovery in Jiangsu Province, China: seedling cloning and transplantation. Ecol. Eng..

